# Neonatal Outcome Associated With Maternal COVID-19 Infection in Adolescent Patients

**DOI:** 10.7759/cureus.29006

**Published:** 2022-09-10

**Authors:** Ana V Uzunov, Diana C Secara, Monica M Cirstoiu

**Affiliations:** 1 Obstetrics and Gynaecology, "Carol Davila" University of Medicine and Pharmacy, Bucharest, ROU; 2 Obstetrics and Gynaecology, University Emergency Hospital, “Carol Davila” University of Medicine and Pharmacy, Bucharest, ROU

**Keywords:** stillbirth, congenital anomalies, low birth weight, preterm birth, adolescent pregnancy, covid-19, neonatal outcome

## Abstract

Background

Coronavirus disease 2019 (COVID-19) infection during pregnancy has been associated with high rates of preeclampsia, stillbirth, and preterm birth. Adolescent pregnancy has also been associated with various adverse maternal and neonatal outcomes, including preeclampsia, stillbirth, preterm birth, congenital anomalies, and low birth weight. Therefore, this study aimed to determine whether COVID-19 infection associated with adolescent pregnancy represents an additional risk factor.

Methods

We performed a study that included 17 adolescent COVID-19- positive patients, who delivered in the Department of Obstetrics and Gynecology of University Emergency Hospital, Bucharest, between 01.04.2020 and 15.04.2022, and a control group of 17 patients who were COVID-19-negative and delivered in the same period in the same unit. In the control group, additional risk factors that could affect neonatal outcomes were excluded. The COVID-19 infection was confirmed using a polymerase chain reaction (PCR) test. The analysis of neonatal outcomes included preterm birth, low birth weight, stillbirth, congenital anomalies, and Apgar score calculated at one minute.

Results

The data from this study showed that COVID-19 infection does not influence the newborn's weight or Apgar score in adolescent patients. Also, in our study, COVID-19 infection was not statistically significant according to preterm delivery in adolescents.

Conclusion

Adolescent pregnancy represents an important health problem associated with a high risk of maternal and neonatal complications. However, COVID-19 infection does not influence neonatal outcomes in this population.

## Introduction

Coronavirus disease 2019 (COVID-19) represents an important health problem that has had a significant impact on every patient worldwide. Studies have shown that pregnant women may have an increased risk of maternal and neonatal complications due to COVID-19 infection [1,2]. However, data on the impact on neonatal outcomes are still inadequate because the majority of published studies have included a limited number of patients [1]. Lam et al. reported that neonatal complications in newborns from mothers infected with severe acute respiratory syndrome coronavirus 2 (SARS-CoV-2) include preterm delivery and small for gestational age [3,4]. Nevertheless, one study reported no higher risk for preterm delivery in pregnant women who were COVID-19 positive [3,5]. Symptoms associated with COVID-19 include fever, cough, fatigue, and dyspnea; however, with every new mutation of the virus, the clinical manifestations may change [3,6]. The same symptomatology has been observed in pregnant women but diarrhea and malaise have also been described [3,7]. Although the actual data are contradictory and limited, most studies have shown that COVID-19 infection may increase the risk of maternal and neonatal complications so the management should be personalized with close maternal and fetal monitoring in a health care facility [8].

 As for adolescent pregnancies, it is known that this is one of the most disputed public health problems especially in developing countries because it has a major impact on maternal and fetal outcomes, but also on education, society, and the next generations. Adolescence is defined as the period of life between childhood and adulthood: between 10 and 19 years of age [9,10]. The World Health Organization (WHO) estimates that approximately 16 million adolescents aged 15-19 years give birth every year and this accounts for 11% of all births all over the world [9,10]. Adolescents are at higher risk for complications associated with pregnancy and childbirth [9]. Patients aged 10-19 years have a higher risk to develop preeclampsia, eclampsia, and systemic infections [9,11]. Newborns from young mothers are also exposed to increased risks for low birth weight, prematurity, stillbirth, congenital anomalies, and early neonatal demise [9,11,12-16]. The explication of these complications may result in the lack of maturation of biological, psychological, and sociological systems [12,16].

As COVID-19 infection has affected patients of all ages worldwide, adolescents have been no exception. Therefore, we analyzed the effect of SARS-CoV-2 infection associated with adolescent pregnancies.

## Materials and methods

We performed an observational, retrospective study that included 17 adolescent patients who were COVID-19 positive and delivered in the Department of Obstetrics and Gynecology of University Emergency Hospital in Bucharest between April 1, 2020, and April 15, 2022, and a control group of 17 randomized adolescent COVID-19-negative patients who delivered in the same period in the same unit. The sample of the study group represented all adolescents who delivered in our unit and were diagnosed with SARS-CoV-2. The data regarding pregnancy, COVID-19 status, and neonatal outcomes were collected from the hospitalization sheets and the Base Data System of the University Emergency Hospital in Bucharest. None of the patients had severe pneumonia caused by SARS-CoV-2 infection. Inclusion criteria were patients aged between 10 and 19 years old who delivered at the University Emergency Hospital in Bucharest and signed themselves or the legal tutor (in case of patients under 16 years old) who signed the informed consent about the study. Exclusion criteria included patients with associated comorbidities that could affect the neonatal outcomes such as infections caused by hepatitis C and hepatitis B viruses, chronic maternal diseases, other respiratory conditions, or refusal to sign the contest.

The age of the mother was defined as it was recorded at the time of delivery. The COVID-19 infection was established by the polymerase chain reaction (PCR) test at the time of hospitalization. Delivery was defined after the fetus completed 24 weeks of gestation or weighed over 500 grams. The neonatal status, including stillbirth, fetal weight, Apgar score calculated at one minute, and congenital anomalies, was evaluated by the neonatology team.

The study was approved by the Ethical Committee of the University Emergency Hospital in Bucharest.

The chi-square test was used to compare the differences between newborns' outcomes from those of adolescent patients with COVID-19 to those without COVID-19.

## Results

**Figure 1:**
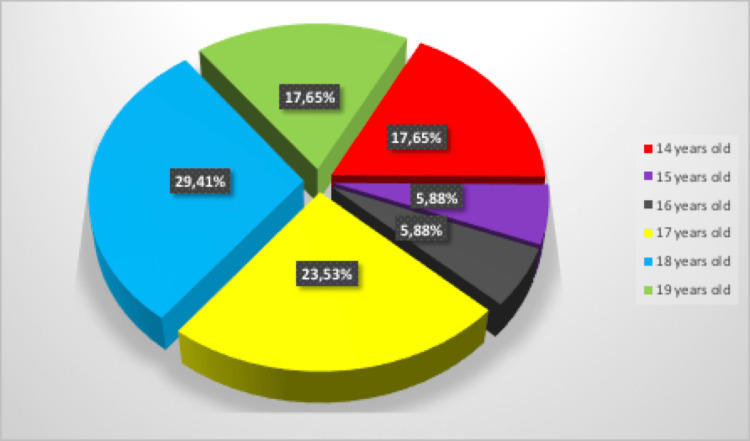
The distribution of COVID-19 infection according to the age of the patients

First, the distribution of COVID-19 infection according to the patient's age was evaluated. The most affected patients by the SARS-CoV-2 virus were the ones aged 18 (n=5) and 17 years (n=4) (Figure 1). In addition, we have to mention that three patients aged 14 years, representing 17,65%, were COVID-19 positive. Regarding patient symptomatology, 88.24% (n=15) of the patients were asymptomatic. The clinical manifestations of the symptomatic patients (n=2) included fever, cough, headache, and rhinorrhea.

**Figure 2:**
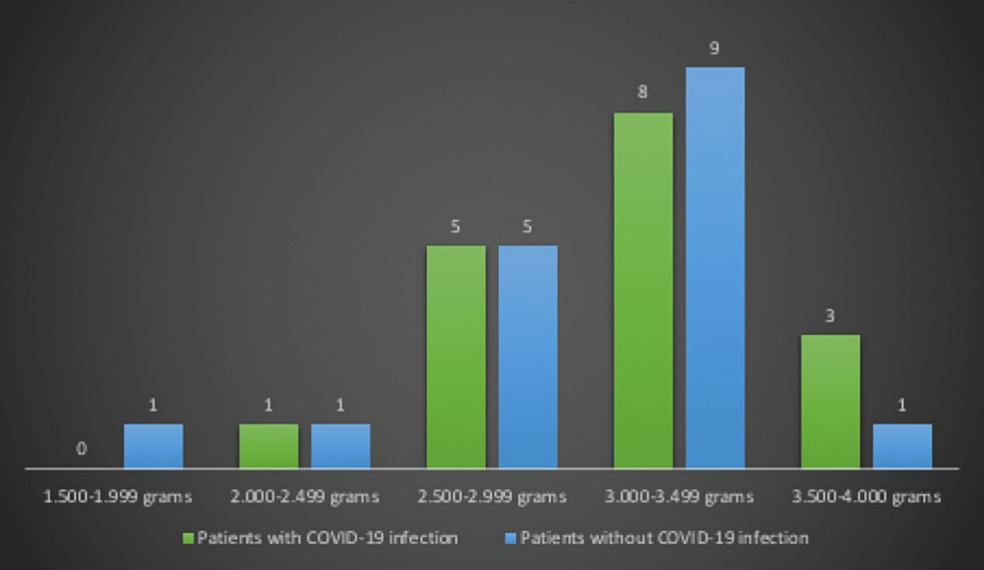
The distribution of the weight of newborns in the two analyzed groups

The birth weights from adolescent mothers with COVID-19 infection were compared with those from adolescent mothers without COVID-19 infection (Figure 2). In the study group, newborns weighed between 2.000 and 4.000 grams while in the control group, newborns weighed between 1.500 and 4.000 grams. Most of the newborns from mothers with SARS-CoV-2 virus (64.71%) weighed over 3.000 grams while in the control group, the percentage was lower (58.82%). We noticed that in both groups, no newborns weighed under 1.500 grams or over 4.000 grams.

The chi-square test was used to compare the newborns' weight in the two groups, but it was not statistically significant (Table 1).

**Table 1:** Chi-square test for the weight of newborns

	Value	p-value
Pearson Chi-Square	2,059	0,725

**Figure 3:**
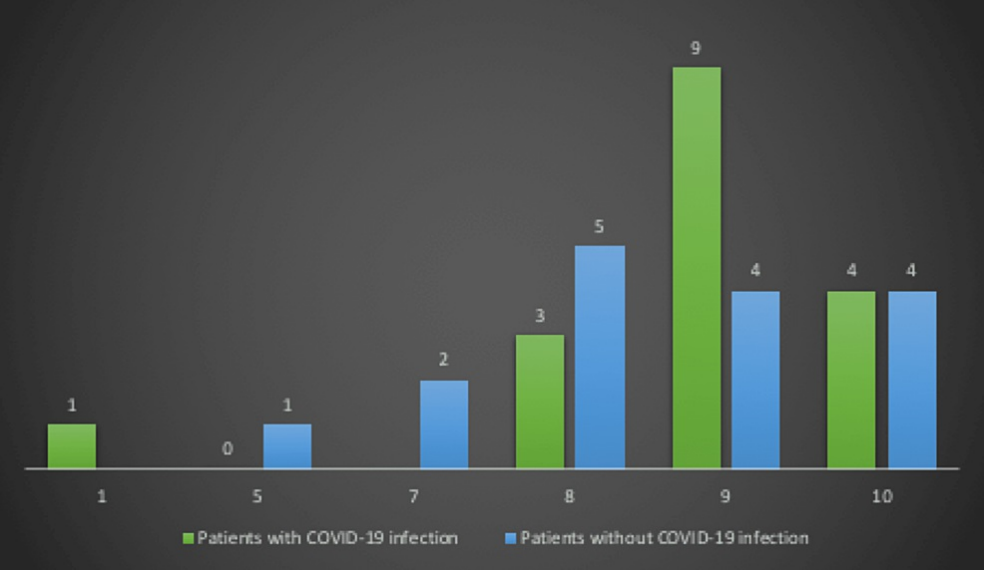
The distribution of the Apgar score calculated at one minute in the two analyzed groups

The neonatal status immediately after birth was evaluated using the Apgar score calculated at one minute (Figure 3). The majority of newborns from mothers infected with COVID-19, representing a percentage of 52.94%, had an Apgar score of 9 and 23.52% had an Apgar score of 10. In this group, only one newborn had an Apgar score of 1. In the control group, 29.41% of newborns had an Apgar score of 8, 23.52% had an Apgar score of 9, and a similar proportion had an Apgar score of 10. The chi-square test showed no statistical significance (Table 2).

**Table 2:** Chi-square test for Apgar score

	Value	p-value
Pearson Chi-Square	2,411	0,299

In the control group, there was one case of stillbirth, whereas no stillbirths were observed in the study group.

Regarding congenital anomalies, there was a newborn from an adolescent patient with COVID-19 infection diagnosed with congenital malformation. None of the newborns included in the control group was diagnosed with congenital anomalies.

As we talk about preterm births, in the study group, there were two newborns delivered before 37 weeks of gestation, representing a percentage of 11.76% while in the control group, there was only one preterm delivery, representing a percentage of 5.88%. The chi-square test was not statistically significant (Table 3).

**Table 3:** Chi-square test for preterm delivery

	Value	p-value
Pearson Chi-Square	0,366	0,545

## Discussion

COVID-19 infection remains a challenge because its physiopathology is not completely understood. As this virus is currently being studied and actual research is limited, we can only suppose that its effects on neonatal status may be similar to those of other viruses and these could be represented by fetal growth restriction, preterm birth, or perinatal mortality [17]. The adverse outcomes of newborns from mothers infected with SARS-CoV-2 are still under discussion. There are studies showing that preterm delivery is higher in patients with COVID-19 infection while data from Ireland and Denmark show that COVID-19 infection did not affect the rates of preterm delivery. Even more, they declined during this pandemic period [17-20]. A large study found an important difference between the neonatal status from mothers who were COVID-19 positive compared to the ones from mothers who were COVID-19 negative [21]. The results showed that newborns from patients with SARS-CoV-2 infection have a higher risk of having low birth weight and lower Apgar scores than newborns from uninfected mothers [21]. As we talk about congenital malformation, Çakırca et al. suggested that no congenital malformations were reported in their study population [22,23]. Congenital malformations were presented as case reports only. As there are no large studies indicating a correlation between SARS-CoV-2 infection and congenital anomalies, it is unclear whether the virus can affect newborn development [22].

According to adolescent pregnancies, this population is known to have a higher risk of neonatal complications such as preterm birth, low birth weight, low Apgar score, and perinatal mortality [12]. The cause of these outcomes can be explained by multiple factors such as poor education, lack of sex education, and poverty [12]. Another possible explanation is the physiological and biological immaturity of these patients.

In our study, we analyzed the impact of SARS-CoV-2 infection on newborns from adolescent patients. Therefore, we compared the neonatal outcomes from newborns from adolescent patients with SARS-CoV-2 infection to those without infection. As previous studies have shown that newborns from adolescents have a higher risk of low Apgar scores or low birth weight, our study found that most newborns from both groups had an Apgar score calculated at one minute higher than 7 and weight higher than 2.500 grams. Regarding the neonatal status from COVID-19-positive patients, we found no evidence of a higher risk of low birth weight nor lower Apgar score compared to those from COVID-19-negative patients. According to the risk of perinatal mortality, there were no cases of demise in the study group while there was one case in the control group. There were no cases of early neonatal death.

Our study found a double percentage of preterm deliveries in the study group compared with the control group, but this was not statistically significant. An important point is that a case of a congenital anomaly was diagnosed in a newborn from a COVID-19 mother while no case of fetal malformation was observed in the control group.

Study limitations

The small number of patients analyzed was the main limitation of this study. The explication results in the fact that SARS-CoV-2 infection affects adolescents less often than in the adult population. Another limitation is that adolescent patients have inadequate antenatal care; therefore, the neonatal status can be influenced by pathologies other than the COVID-19 virus or the one we can diagnose at the time of delivery.

## Conclusions

Adolescent pregnancies complicated by COVID-19 infection require special attention because adverse neonatal outcomes can have serious implications. The actual study did not show that COVID-19 infection is an additional risk factor for newborns from adolescent mothers, but as this population is at high risk for fetal complications, every medical team should be aware. Additionally, more educational programs should be created for the prevention of pregnancy during adolescence and for the prevention of COVID-19 infection during pregnancy.
